# Sangiovese *cv* Pomace Seeds Extract-Fortified Kefir Exerts Anti-Inflammatory Activity in an *In Vitro* Model of Intestinal Epithelium Using Caco-2 Cells

**DOI:** 10.3390/antiox9010054

**Published:** 2020-01-08

**Authors:** Gabriele Carullo, Paolo Governa, Umile Gianfranco Spizzirri, Marco Biagi, Fabio Sciubba, Gianluca Giorgi, Monica Rosa Loizzo, Maria Enrica Di Cocco, Francesca Aiello, Donatella Restuccia

**Affiliations:** 1 Dipartimento di Farmacia e Scienze della Salute e della Nutrizione—Dipartimento di Eccellenza 2018-2022, Università della Calabria, Edificio Polifunzionale, 87036 Rende (CS), Italy; gabriele.carullo@unical.it (G.C.); umile_gianfranco.spizzirri@unical.it (U.G.S.); monica_rosa.loizzo@unical.it (M.R.L.); donatella.restuccia@unical.it (D.R.); 2 Dipartimento di Biotecnologie, Chimica e Farmacia—Dipartimento di Eccellenza 2018-2022, Università di Siena, Via Aldo Moro 2, 53100 Siena, Italy; paolo.governa@unisi.it (P.G.); gianluca.giorgi@unisi.it (G.G.); 3 Dipartimento di Scienze Fisiche, della Terra e dell’Ambiente, Università di Siena, Via Laterina 8, 53100 Siena, Italy; biagi4@unisi.it; 4 Dipartimento di Chimica, Università di Roma “La Sapienza”, Piazzale Aldo Moro 5, 00185 Roma, Italy; fabio.sciubba@uniroma1.it (F.S.); mariaenrica.dicocco@uniroma1.it (M.E.D.C.)

**Keywords:** wine pomace, antioxidant activity, TEER, Caco-2 cell line, milk kefir, phenol red, polyphenols, extraction

## Abstract

Inflammatory bowel disease and food allergies are a growing topic in the field of nutrition science. Polyphenols, which are the most important secondary metabolites of plants, demonstrated to modulate the expression and/or production of numerous proteins, but also to regulate the intestinal ecosystem. In this context, our aim was the investigation of protective effects against the gastrointestinal mucosa of fortified milk kefir obtained by adding seeds extract from Sangiovese *cv*. Pomace. Methods: An ultrasound-assisted method was used to obtain the extracts. All the extracts were assayed for the antioxidant activity. The best extract was used as an additive of fermented milk kefir to obtain a fortified final product. Kefir samples were analyzed by NMR spectroscopy. The efficiency of the barrier functions was evaluated by measuring trans-epithelial electric resistance (TEER) using a voltmeter. Results: the enriched kefir (Ksgn) possesses higher antioxidant performances compared to the unfortified sample (Kwht). Kwht and Ksgn did not alter Caco-2 TEER in basal condition.

## 1. Introduction

The gastrointestinal mucosa represents one of the most important barrier of the human body. It is constituted of several layers: the mucus layer, with immune-sensing and regulatory proteins; the intestinal epithelial monolayer; the tight junctions, located at the apical side of the cells, with the aim to regulate the transport of small molecules and ions; the adherens junctions and desmosomes, responsible of the maintenance of the integrity of the intestinal barrier; the lamina propria, containing immune cells (e.g., T cells, B cells, macrophages, and dendritic cells) which take part in the immunological defense mechanisms of the intestinal barrier [1]. The gastrointestinal mucosa has two main important functions: it is a filter with selective penetrability, allowing the transit of nutrients from the intestinal lumen into the blood stream; it is also a barrier, preventing the permeation of dangerous entities such as microorganisms, antigens, and pro-inflammatory factors. Indeed, microorganisms are normally present in the healthy intestine; however, because of several dysfunctions or diseases, this barrier could be assaulted by pathogens and physical stimuli provoking hyper-permeability, which is supposed to contribute to the pathogenesis of several gastrointestinal disorders together with inflammatory bowel disease, food allergy, and celiac disease [2]. The maintenance of intestinal permeability as a feature for overcoming the development of severe diseases, including inflammatory bowel disease and food allergies, is a growing topic in the field of nutrition science. Various substances have been used over the years, [3,4,5] including the conventional steroid drugs, able to improve gut health through the reduction of inflammatory mediators [6]. Polyphenols, which are the most important secondary metabolites of plants, demonstrated to modulate the expression and/or production of numerous proteins, but also to regulate the intestinal ecosystem [7]. Indeed, the modulation effect on gut microbiota has been reported for polyphenols such as curcumin, quercetin, shogaol, and rosmarinic acid, but also tannins and anthocyanins, which could be useful in the management of gastrointestinal disorders [8]. Anthocyanins and anthocyanin metabolites are useful in improving intestinal barrier function; indeed, these bioactives molecules modulate oxidative stress and inflammation directly in intestinal epithelial cells, but their low bioavailability limited their use as functional foods. In addition, red wine polyphenols can reinforce and protect the intestinal barrier against inflammatory stimuli by affecting the tight junction protein expression [9]. Noteworthy, not only plant-derived polyphenols are able to support the gastrointestinal barrier, but also polyphenols derived from milk fermentation, which can be found in products such as yogurt and kefir [10,11]. Fermented milk products showed protective effects towards the intestinal barrier. Indeed, milk fermented with *Lactobacillus fermentum* was able to ameliorate indomethacin-induced inflammation in the gastrointestinal tract [12]. Combinations of *Lactobacillus* spp., *Bifidobacterium* spp., and *Streptococcus salivarius* decreased the initial onset of intestinal inflammation, resulting in protective effects mediated by different mechanisms. In the acute pancreatitis, a severe inflammatory condition, probiotic’s pre-treatment prevented tight junction protein disruption via glutathione biosynthesis [13]. Milk fermented with *Lactobacillus casei* demonstrated to reduce the expression of TNF receptor 2 (TNFR2), myosin light-chain kinase (MLCK), leading to the restoration of physiological gastric condition after inflammatory stimuli [14]. Among the fermented milk products, kefir is now becoming popular with consumers and new producing companies are rising worldwide. Kefir is a fermented milk beverage originating from the Balkans and Caucasus with acidic-alcoholic taste and natural carbonation. Its microflora is primarily composed by lactic acid bacteria (*Lactobacillus*, *Leuconostoc*, *Lactococcus*, and *Streptococcus* spp.), yeasts (*Saccharomyces* and *Kluyveromyces* spp.) and acetic acid bacteria (*Acetobacter* spp.), even if many differences can be found depending on the product origin and processing [15]. Kefir bacterial and fungal species live in symbiosis in the grains, which are closed matrices composed by microbial derived proteins and polysaccharides (mainly kefiran). Kefir grains are directly added to milk and, once the fermentation has occurred, they are removed to obtain the beverage. Kefir is now widely accepted as a powerful probiotic source to improve gut health, with positive nutritional attributes related to its content of carbohydrates, proteins, vitamins, antioxidants, and minerals [16]. Other compounds produced during the activity of microflora include organic acids (i.e., lactic, acetic, pyruvic, propionic, and butyric acid), flavor molecules (i.e., diacetyl and acet-aldehyde), exopolysaccharides (i.e., kefiran), as well as antibiotics and numerous types of bactericide [17]. However, the antimicrobial and antibacterial activities are only two of the numerous health promoting effects associated with kefir consumption. In fact, many other benefits are ascribed to kefir, including effect on immune system, anti-inflammatory activity, hypocholesterolemic function, β-galactosidase activity, gastrointestinal proliferation, bacterial colonization, anti-diabetic features, anti-allergic and anti-oxidative properties, effect on blood pressure and lipid level and protection against apoptosis [18]. In this field, kefir resulted also an important anti-inflammatory tool, due to the presence of *Lactobacilli* [19]. Little information is available on the influence of polyphenol-containing extracts on the kefir beverage. Two different studies evaluated the synergy between wine by-products (prebiotic) and the kefir microflora (probiotic). Cho and colleagues (2018) investigated the influence of a diet enriched with wine grape seed flour (GSF) and kefir lactic acid bacteria (LAB) on high-fat diet-fed obese mice [20] demonstrating the synergistic action of GSF and LAB against high fat-induced obesity and inflammation. The same research group assessed the probiotic characteristics (i.e., artificial gastric and intestinal resistance, cholesterol reduction, and adherence of yeast to Caco-2 cells) and antioxidant activity of four strains of the kefir yeast, *Kluyveromyces marxianus*, in combination with GSF and/or with a grape seed extract (GSE) [21]. Among the evaluated microorganisms, it was found that *K. marxianus* KU140723-02 showed the best antioxidant properties, doubled when incubated with GSF/GSE. Despite these promising data, to the best of our knowledge, no data reporting the effect of an enriched kefir on intestinal inflammation is available. Thus, the aim of this study was to prepare a kefir, starting from Kefiralia grains, skimmed UHT milk and a polyphenol-containing extract deriving from grape pomace (*cv.* Sangiovese), in order to evaluate the antioxidant and anti-inflammatory profile using Caco-2 cell line as a model of intestinal epithelium.

## 2. Materials and Methods

### 2.1. Chemicals and Reagents

All chemicals and reagents used in this study were purchased from Merck (Darmstadt, Germany) and VWR International (Milan, Italy) and, unless specified otherwise, were of analytical grade or higher. All solvents and standards were purchased from Merck (Darmstadt, Germany). Kefir grains were obtained from Burumart Commerce S.L. (Arrasate, Gipuzkoa, Spain).

### 2.2. Preparation of the Grape Extracts

Skins or milled seeds (30 g), obtained by manual separation from Sangiovese pomace, were added to 200 mL ethanol/water (50/50 *v*/*v*) (pH = 2.0) and the mixture was sonicated at 30 °C (10 cycles/sec) for 15 min, at an ultrasonic frequency of 40 kHz using the ultrasound-bath Branson model 3800-CPXH (Milan, Italy). The mixture was then filtered and concentrated at reduced pressure using a Rotavapor Büchi RII (Cornaredo, Italy). The extracts obtained from skins (sgnbs) and seeds (sgnss) were stored at −18 °C until analyses [22].

### 2.3. Preparation of Kefir 

10 g of kefir grains (Kefiralia) were added in a glass flask at room temperature, containing 50 mL of ultra-high temperature (UHT) skimmed milk. The container was closed non-hermetically and was incubated at 20–25 °C for 24 h with or without extracts addition. The kefir grains obtained (Kwht) were weighted and the pH value measured by using the pH 211 Microprocessor pH Meter (HANNA instruments). The enriched kefir (Ksgn) was obtained mixing 10 mg of the sgnss with 10 mL of Kwht in the same experimental conditions [23].

### 2.4. Electrospray Ionization (ESI) Mass Spectrometry Analysis

The extracts were dissolved in methanol and injected (flow rate 5 μL min^−1^) in the electrospray source of a LCQ DECA ion trap (ThermoFinnigan, Bremen, Germany) and a Orbitrap Q Exactive Plus (Thermo Fisher) at resolution of 30,000 and 140,000 FWHM@m/z 200, in positive and negative ion mode. MS product ion spectra has been resolved in the ion trap with helium as collision gas, and higher energy collisional dissociation (HCD) MS/MS spectra in the Orbitrap Q Exactive Plus by using nitrogen as collision gas at collision energy 18–30% arbitrary units. Phenol-Explorer 3.5 (French National Institute for Agricultural Research) and ChemSpider (Royal Society of Chemistry) have been used as tools for compounds identification [24].

### 2.5. NMR Analysis

Aliquots of kefir samples were analyzed by NMR spectroscopy in order to assess their chemical composition. In particular, the assignment of the resonances was performed by analyzing ^1^H characteristics and cross-correlated signals in 2D ^1^H-^1^H TOCSY spectra and by comparison with literature data [22,25,26]. Quantification of the identified compounds was performed by comparison of the signal integral with the reference one, and quantities were expressed in mg of compound normalized for the aliquot weight expressed in g. Each dry aliquot was dissolved in 0.6 mL of D_2_O:CD_3_OD (2:1 ratio) containing 3-(trimethylsilyl)-propionic-2,2,3,3-d_4_ acid sodium salt 2 mM as chemical shift and concentration reference. All spectra were recorded at 298 K on a Bruker AVANCE III spectrometer operating at the proton frequency of 400.13 MHz and equipped with a Bruker multinuclear z-gradient inverse probehead. ^1^H spectra were acquired employing the *presat* pulse sequence for solvent suppression with 128 transients, a spectral width of 6000 Hz and 64 K data points for an acquisition time of 5.45 s. The recycle delay was set to 6.55 s in order to achieve complete resonance relaxation between successive scansions. ^1^H–^1^H TOCSY experiments were acquired with spectral width of 6000 Hz in both dimensions, a data matrix of 8K × 256 points, mixing time of 110 ms and relaxation delay of 2 s.

### 2.6. Quantification of Total Phenolic Equivalent (Tpe) by Folin–Ciocalteu Procedure 

The amount of total phenolic equivalents (TPE) in the extracts and fortified and unfortified kefir was determined using Folin–Ciocalteu reagent procedure [27]. Total phenolic compounds was expressed as milliequivalents of gallic acid (GA) per grams of extract. In a standard procedure, 6.0 mL of hydro-alcoholic solution (50/50 *v*/*v*) of each sample was placed in a volumetric flask (10 mL) and then Folin–Ciocalteu reagent (1.0 mL) was added and the contents of flask were mixed thoroughly. After 3 min, 3.0 mL of Na_2_CO_3_ (2%) were added, and then the mixture was allowed to stand for 2 h with intermittent shaking. The absorbance was measured at 760 nm using a hydro-alcoholic mixture (50/50 *v*/*v*) prepared under the same reaction conditions as a control. The amount of total phenolic groups in the extracts was expressed as gallic acid equivalent concentrations, by interpolating data with the calibration curve. Obtained using gallic acid as the reference standard [28]. Each measurement was performed in triplicate and data expressed as means (± SD). UV-Vis absorption spectra were recorded with a Jasco V-530 UV/Vis spectrometer (Jasco, Tokio, Japan). 

### 2.7. Determination of Total Antioxidant Capacity (Tac)

The total antioxidant capacity (TAC) of each extract and fortified and unfortified kefir was evaluated as previously reported [29], with some changes. In a standard procedure, 0.3 mL of hydro-alcoholic solution (50/50 *v*/*v*) of each sample were mixed with 1.2 mL of phosphomolybdate reagent solution (0.6 M H_2_SO_4_, 28.0 M Na_3_PO_4_, and 4.0 M (NH_4_)2MoO_4_). The reaction mixture was incubated at 95 °C for 150 min and, after cooling to room temperature, the absorbance of the mixture was measured at 695 nm against a control prepared in the same conditions. Measurement was carried out in triplicate and data expressed as means (± SD). The total antioxidant activity of each extract and fortified and unfortified kefir was expressed as catechin equivalent concentration. By using five different antioxidant standard solutions, a calibration curve was recorded. A volume of 0.3 mL of each solution was mixed with 1.2 mL of phosphomolybdate reagent solution to obtain the final concentrations of 8.0, 16.0, 24.0, 32.0, and 40.0 μM, respectively. After 150 min of incubation at 95 °C, the solutions were analyzed by using a UV-Vis spectrophotometer, and the correlation coefficient (R^2^), slope, and intercept of the regression equation obtained by the method of least-squares were calculated. Each measurement was performed in triplicate and data expressed as means (± SD).

### 2.8. Determination of Scavenging Activity on DPPH Radicals

Free radical scavenging properties of extracts and fortified and unfortified kefir were estimated towards DPPH (2,2-diphenyl-1-picrylhydrazyl)acid)) radical [30]. Next, 1.0 mL of hydro-alcoholic solution (50/50 *v*/*v*) of each sample was placed in a volumetric flask (10 mL) and then 4.0 mL of hydro-alcoholic solution (50/50 *v*/*v*) and 5.0 mL of ethanol solution of DPPH (200 μM) were added, obtaining final DPPH concentration of 100 μM. The sample was incubated in a water bath at 25 °C and, the absorbance of the remaining DPPH was determined colorimetrically at 517 nm after 15 min. The scavenging activity of the tested matrices was measured as the decrease in absorbance of the DPPH and it was expressed as percent inhibition of DPPH radicals calculated according the following Equation (1):

(1) $$inibhition\%=\frac{A_{0}-A_{1}}{A_{0}}\times 100$$

where *A*_0_ is the absorbance of a standard that was prepared in the same conditions, but without any sample, and *A*_1_ is the absorbance of the analyzed samples. The scavenger ability of the sample toward DPPH specie was expressed as IC_50_ value [31]. Each measurement was carried out in triplicate and data expressed as means (± SD).

### 2.9. Determination of Scavenging Effect on the ABTS Radical Cation

Free radical scavenging properties of extracts were estimated towards ABTS (2,2′-azino-bis (3-ethylbenzothiazoline-6-sulphonic acid)) radical [32]. ABTS was dissolved in water at a 7.0 mM concentration. ABTS radical cation (ABTS•+) was produced by reacting ABTS stock solution with 2.45 mM potassium persulfate (final concentration) and allowing the mixture to stand in the dark at room temperature for 12–16 h before use. In order to evaluate the scavenging effect of extracts, 500 μL of hydro-alcoholic solution (50:50 *v*/*v*) of each sample were mixed with 2.0 mL of the ABTS radical solution. The obtained mixture was incubated in a water bath at 37 °C and protected from light for 5 min. The decrease of absorbance at 734 nm was measured at the endpoint of 5 min. The antioxidant activity was expressed as a percentage of scavenging activity on the ABTS radical according to Equation (1). The scavenger ability of the extracts towards ABTS radical was expressed as IC_50_ value. All samples were assayed in triplicate and data expressed as means (± SD).

### 2.10. Cell Culture

Caco-2 cells were used as a stable *in vitro* model for the intestinal epithelium [33]. Cells were a kind gift by Prof. Monica Montopoli (University of Padua, Padua, Italy) and were cultured in 75 cm^2^ cell culture flask (Sarstedt, Milan, Italy) in DMEM supplemented with 10% fetal bovine serum (FBS), 1% glutamine and 1% penicillin/streptomycin antibiotic (Sigma-Aldrich, Milan, Italy) [34,35]. THP-1 monocytes from the European Collection of Authenticated Cell Cultures were purchased from Sigma-Aldrich (Italy) and cultured in 75 cm^2^ cell culture flask in RPMI supplemented with 10% fetal bovine serum (FBS), 1% glutamine, 1% penicillin/streptomycin antibiotic, and 0.05 mM 2-mercaptoethanol (Sigma-Aldrich, Italy). For the inflammatory model, THP-1 cells (1 × 10^6^/mL) were seeded in 75 cm^2^ cell culture flask in Hanks’ Balanced Salt solution (HBSS, Sigma-Aldrich) with 10 mM 4-(2-hydroxyethyl)-1-piperazineethanesulfonic acid (HEPES) and 10 mM D-glucose (pH = 7.4) and incubated with 250 ng/mL lipopolysaccharide (LPS) from *Salmonella enteridis* (Sigma-Aldrich, Italy) for 24 h. The culture medium was then collected and centrifuged and the supernatant was used for the experiments. Untreated cells medium was used as control. Cells were maintained under a humidified atmosphere of 5% CO_2_, at 37 °C.

### 2.11. Measurement of Trans-Epithelial Electric Resistance

The efficiency of the barrier functions was evaluated by measuring trans-epithelial electric resistance (TEER) using a voltmeter [36]. Caco-2 cells (8 × 10^5^) were placed in transparent polyester membrane cell culture inserts with 0.4 μM pore size (Sarstedt) as previously described [37]. Culture medium was replaced every other day. The integrity of the cell monolayers was monitored by measuring the trans-epithelial electric resistance (TEER) of the monolayer from day 14th to day 21st after seeding. When a stable value was reached, a pre-treatment of 24 h was done adding Kwht and Ksgn in the apical chamber at different concentrations (10, 1, and 0.1 µg/mL) in appropriate wells and TEER was measured after 0, 4, 8, and 24 h. TEER measurements were performed in HBSS with 10 mM Hepes and 10 mM D-glucose (pH = 7.4), after an equilibration period at room temperature [38,39]. Only cells with TEER value within 360–500 Ω × cm^2^ were used for the experiments [40,41,42]. After 24 h of pre-treatment, the basal chamber medium was replaced with LPS-stimulated THP-1 medium and TEER was measured after 0, 4, 8, and 24 h. Millicell^®^ ERS meter, Millipore Corporation (Bedford, MA, USA) connected to a pair of chopstick electrodes was inserted in the apical and basolateral chambers. TEER was expressed as percentage of resistance, normalized to initial value.

### 2.12. Paracellular Permeability Assay

Phenol red flux across Caco-2 cell monolayers was used as a measure of paracellular permeability. At the end of the TEER experiment, the apical medium was replaced with a 500 mM solution of phenol red (Sigma-Aldrich, Italy) in HBSS and the basolateral medium was replaced with fresh HBSS. After 60 min of incubation at 37 °C, 100 μL was collected from the basolateral chamber and added to 10 μL of 1N NaOH in 96 multiwell plates. Phenol red leakage was measured using a SAFAS MP96 spectrophotometer (Safas, Principality of Monaco) by recording the absorbance at 540 nm, and quantified according to a calibration curve obtained with serial dilutions (500–0.5 mM) of phenol red solution in HBSS [43,44,45].

### 2.13. Statistical Analysis

All experiments were performed in duplicate in three independent replicates. A D’Agostino and Pearson test was used to check the normal distribution of the data. The statistic differences between groups were determined by the analysis of the variance (ANOVA). Values are expressed in the range of +/− standard deviation and *p* < 0.05 was considered statistically significant. Graphs and calculations were performed using GraphPrism^®^ (GraphPad, La Jolla, CA, USA).

## 3. Results and Discussion

### 3.1. Extraction of Grape Pomace

The extraction method used is a green method, able to give back a good amount of extract in small time. Skins and seeds, after separation, were triturated and extracted without any further washing. Ethanol was of pharmaceutical grade, in order to reduce the use of organic solvents that can limit the employ of extract in food supplement. 

### 3.2. Evaluation of Antioxidant Activity of Extracts

Wine pomace has been extensively described as an important source of phenolic compounds, with notable qualitative and quantitative differences [46]. Basically, grape compositions and enological practices can be considered the two main factors that directly affect the phenolic profile during the winemaking process [47]. Folin–Ciocalteu method was proposed in the determination of the disposable phenolic equivalents (TPE) of the grape skins and seeds from Sangiovese *cv*. This simple and reproducible procedure is based on the electrons that are transferring from phenolic compounds to the Folin–Ciocalteu reagent in alkaline medium. The TPE values of sgnbs and sgnss are given in Table 1 and clearly showed that raw material (seeds or skins) drastically influenced the phenolic content of the extracts. 

In general, lower amounts of phenolic compounds were detected in sgnbs (0.549 meq GA/g of extract), compared to the sgnss (1.620 meq GA/g of extract). These data are consistent with those obtained by Tang and colleagues [48] who found higher TPE value in the seed compared to the peel of grape wastes of Chinese and Australian autochthones cultivars, with TPE values being almost one order of magnitude higher than our data. This finding can be explained by considering the differences concerning the grape cultivar and the agronomic conditions of the grape production country. TPE values are often, but not always, related to the antioxidant activity of the extract because of other molecules, such as terpenes and sterols, possessing relevant antioxidant behavior [49]. In our study, the total antioxidant capacity (TAC) and scavenging activity in aqueous and organic environment (Table 1) highlighted a good correlation between TPE and antioxidant capacity. This data was confirmed by mass spectrometry (ESI-MS) analyses, displaying the presence of a great variety of molecules bearing phenolic rings, to which the radical scavenging activity may be attributed [50]. The scavenger activity toward lipophilic DPPH radical was expressed as the IC_50_ (mg mL^−1^), as reported in Table 1. A comparison of IC_50_ values shows that seeds extracts lead to IC_50_ values one order of magnitude lower than skins extracts (0.160 mg mL^−1^ for sgnbs compared to 0.012 mg mL^−1^ for sgnss). The data showed a quite good correspondence with the results of TAC and TPE. This trend is evident for both the samples and some discrepancies could be justified by considering the different environment (organic and aqueous) in which the assays were performed. The scavenging capacity of the extracts in the aqueous environment against the ABTS radical was expressed in terms of IC_50_ (mg mL^−1^), and Table 1 displayed the recorded data. The analysis of the IC_50_ values exhibited an antioxidant capacity of the peel extracts more than one order of magnitude higher that the extracts obtained from the seeds confirming the data observed in the evaluation of scavenging activity in the organic environment against DPPH radical. However, the scavenging activity recorded in the aqueous environment appears more than three times higher compared to the organic one. Otherwise, literature data confirmed that ABTS assay represents more accurately the antioxidant capacity of vegetable matrices containing hydrophilic and lipophilic molecules [51]. Lack of data concerning extracts antioxidant capacity of the investigated cultivars made the comparison of the collected values with the literature analyses very difficult. In addition, many factors, such as fruit ripening, weather conditions, soil and place of growth, largely affected the distribution of the antioxidants in the vegetable matrix further complicating whatever qualitative-quantitative comparison. The sgnss was selected to perform kefir preparation. As reported in Table 2 and Table 3, the substances that should be responsible of the antioxidant profile are peonidin glucoside, malvidin-3-O- glucoside, malvidin cumaroyl glucoside, but also methyl glucuronic acid, ethyl glucuronic acid, hydroxymethyl monoglycosylpyranosonic acid and ferulic acid. [52,53]

### 3.3. Kefir Preparation and NMR Analysis

**Figure 1:**
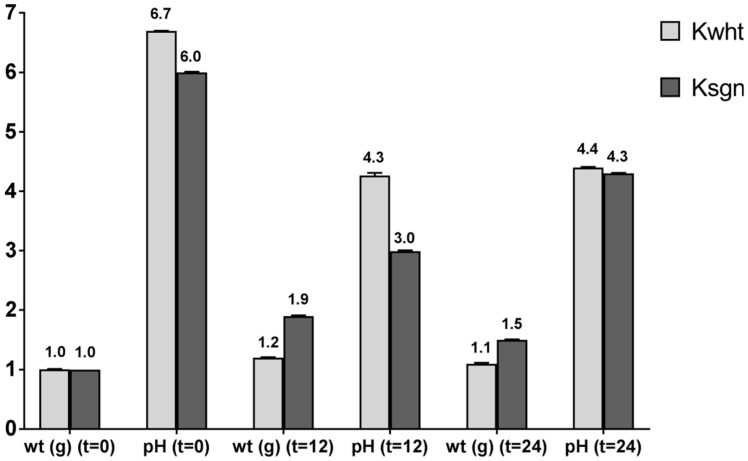
Kefir growth during fermentation.

In our procedure, kefir was prepared by adding the sgnss extract before the fermentation. The growth was maintained for 12 and 24 h. pH levels and weight of grains have been evaluated. As reported in Figure 1, pH levels were similar at t = 0, while after 12 h they were lower (pH = 3.0) in Ksgn compared to Kwht. Nevertheless, after 24 h, which is the usual fermentation time for kefir growth, pH levels were much higher, but still in the medium acidic range. The weight of grains increased considerably after 12 h in Ksgn, with a decrease after 24 h. No significant increase has been observed in Kwht. 

The metabolites determined by ^1^H NMR techniques in the control and fortified kefir are reported in Table 4. As can be seen, the same amino acids, carbohydrates and organic acids composition was found in both Ksgn and Kwht, suggesting that, at the fortification level employed (i.e., 0.0162 meq/GAE), the extract did not affect the end-products of the microbiota metabolic pathways. However, different concentrations have been found for some molecules (mainly carbohydrates, alanine, phenylalanine, succinic, and citric acid) meaning that the presence of the extract influenced the rate and/or the route of formation/accumulation of some metabolites from a quantitative point of view. Further investigations would be useful to evaluate the extent by which the extract addition may influence the kefir composition. As can be noted, most of the compounds found in sgnss were not detected in kefir samples, demonstrating their conversion into other chemical species by microbial metabolism. In particular, the chosen level of supplementation allowed reaching the proper kefir functionalization avoiding, at the same time, possible negative effects.

Indeed, several limitations were reported when using wine by-products extracts for dairy fortification, with the main issues being related to the antimicrobial effect of such extracts on LAB, as well as their negative impact on sensory and rheological features of the functional product [54,55,56,57]. 

It is well documented that kefir microorganisms include homo- and hetero-fermentative LAB (mainly *Lactobacillus*, *Lactococcus*, *Leuconostoc*, and some *Acetobacter* spp.) together with lactose- and non-lactose-assimilating yeast (mainly *Saccharomyces*, *Kluyveromyces*, and *Candida)*. Interestingly, ethanol was never found in our samples, indicating that only homo-fermentation took place during the kefir preparation. This is not surprising, as wide variations in ethanol concentrations have been reported, depending on grain origin and production methods [16]. In control kefir, lactose was totally degraded to galactose and glucose by kefir microbiota. On the contrary, the fortified sample still contained lactose (2.26 g L^−1^), although glucose and galactose concentrations were higher (8.31 g L^−1^ and 10.72 g L^−1^, respectively) compared to the non-fortified counterpart (4.22 g L^−1^ and 3.65 g L^−1^, respectively). The last aspect could be related to the production of kefiran by homo-fermentative LAB. This extracellular exopolysaccharide, is a hetero-polysaccharide made of glucose and galactose in equal amounts and forming the major portion of the gelatinous matrix containing the kefir microflora [58]. As kefiran was removed during the beverage sieving, the control sample showed lower amounts of glucose and galactose, while in the fortified sample, the process of kefiran production seemed to proceed slower and/or to a lesser extent, favoring the production of lactic acid. The latter, as expected in a homo-fermentative process, was the highest in concentration among the detected organic acids in both samples (1.48 g L^−1^ and 2.61 g L^−1^, control and functional kefir, respectively). The production of other organic acids, including acetic, formic, citric and succinic has been also reported at very different concentrations depending on kefir production process [59,60]. In this work, for the first time, we also detected the 2-idroxy-isobutyric acid. All organic acids are very important for the aroma of the final product as well as for its preservation, as they reduce the development of undesirable or pathogenic microorganisms, due to their effect on acidity rising [61]. Low quantities of pyruvic acid were expected, as pyruvate is the substrate for some kefir microorganisms (i.e., *Lactobacillus lactis*, L. *acidophilus*, *Streptococcus thermophilus*, and *Kluyveromyces marxianus*) to produce lactic acid by the Embden–Meyerhof–Parnas (EMP) pathway. Some organic acids can be further converted in volatile flavor compounds. Residual pyruvate, in fact, can form acetaldehyde and diacetyl, while citrate is the preferred substrate for acetoin and diacetyl production by some LAB. In particular, the high concentration of citric acid in fortified kefir (1.06 g L^−1^) could indicate an incomplete metabolism of this compound, also confirmed by the low level of succinic acid (2.83 mg L^−1^), an intermediate of the citrate metabolism [62]. Anyway, it may reasonably be supposed that the presence of many organic acids in the grape seed extract, could have influenced metabolite profiles and distributions in fortified sample. As far as amino acids are concerned, only alanine, valine, isoleucine, tyrosine and phenylalanine were detected in kefir samples. Similarly to acetic acid bacteria and yeasts, Lactococci have been reported to induce proteolytic activity in kefir [60,61]. Guzel-Seydim and co-workers [63] demonstrated the formation of serine, lysine, alanine, and threonine in kefir, while Liut Kevicius and Sarkinas (2004) [64] showed valuable concentrations of tryptophan, valine, lysine, methionine, phenylalanine, threonine, and isoleucine in similar samples. Also in this case, wide variations of quantities and distributions can be found, depending on grain origin and kefir production.

### 3.4. Antioxidant Activity of Kefir and Fortified Kefir

Polyphenols and their metabolites act as activators/inhibitors of bacterial growth depending on their concentration and chemical structure [65]. Growing evidence confirm their ability to influence the microbiota composition through the inhibition of pathogen growth and the stimulation of the commensal bacteria growth [66]. During fermentation, enzymes such as β-glycosidase determine the hydrolysis of complex phenolic compounds to simpler molecules and increase in quantitative amount of TPE while lactic acid bacteria or other microorganisms can increase the level of total phenolic content [67]. This improving can be expanded by adding a source of active molecules before the fermentation process. In fact, as phenolic compounds present in sgnss are able to interact with milk proteins, these may further affect the sensory and functional properties, as well as microbiological quality and oxidative stability, of the dairy products. The results of antioxidant activity (TAC and DPPH free radical scavenging assays) showed that Ksgn had higher antioxidant activity compared to Kwht (Table 5).

Specifically, the addition of the extract displayed an increase of of TAC value by 40.7%, while IC_50_ value against DPPH radical highlighted a decrease by 16.0%, compare to control kefir, clearly showing that enriched kefir possess higher antioxidant performances compared to the unfortified sample. This finding is consistent with those reported in the literature, highlighting a positive correlation between vegetables-enriched fermented products and antioxidant activity [68]. The antioxidant properties of the fortified kefir are in accord with the concentrations of TPE, confirming that the phenolic compounds, which can scavenge free radical and active oxygen species, have a critical importance in determining the antioxidant capacity of foods. In our experiments, TPE value significantly increased from 1.615 to 1.975 meq GA per L, as a consequence of the addition of sgnss extract to the unfermented food matrix before the kefir production [69].

### 3.5. Kwht and Ksgn Protect Intestinal Barrier from Inflammatory-Impaired Permeability

**Figure 2:**
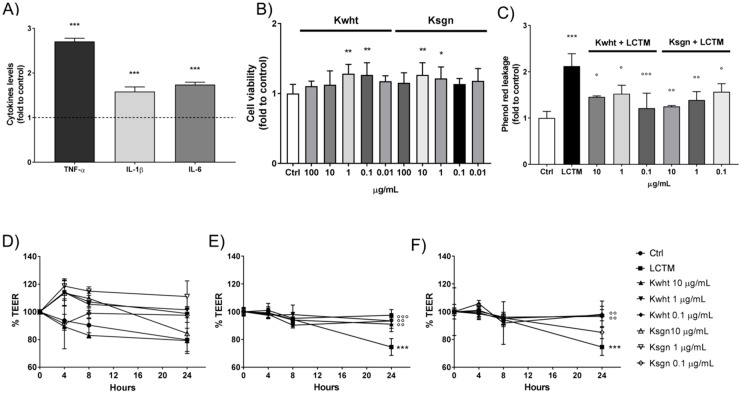
Effects of KL5 and KL5E10 in the *in vitro* model of inflamed intestinal epithelium. (**A**) LPS-stimulated THP-1 cytokines release normalized to control (dashed line); (**B**) Caco-2 cell viability with different concentrations of the samples; (**C**) phenol red permeability assay; (**D**) effect of the samples on Caco-2 trans-epithelial electric resistance (TEER) in basal conditions; (**E**) effects of Kwht on Caco-2 TEER in inflammatory conditions; (**F**) effects of Ksgn on Caco-2 TEER in inflammatory conditions. * *p <* 0.05 vs. Ctrl; ** *p <* 0.01 vs. Ctrl; *** p < 0.001 vs. Ctrl; *° p* < 0.05 vs. stimulus; *°° p* < 0.01 vs. stimulus; *°°° p* < 0.001 vs. stimulus.

Intestinal epithelium dysfunctions are related to strong antigenic response which lead to the onset of inflammation and damages to the intestinal barrier with increased permeability [70,71,72,73,74]. In this *in vitro* model, we reproduced the inflammatory-impaired intestinal barrier by stimulating Caco-2 cells with LPS-conditioned THP-1 medium (LCTM). Indeed, LPS caused a marked release of pro-inflammatory cytokines from THP-1 cells, which significantly reduced LCTM-stimulated Caco-2 monolayer TEER values by approximately 25%, compared to the untreated control, after 24 h. Consistently, phenol red permeability was approximately two-fold higher in inflamed Caco-2, compared to the untreated control. Kwht and Ksgn did not alter Caco-2 TEER in basal condition. On the contrary, Kwht totally counteracted the cytokines-impaired TEER and phenol red permeability at each of the tested concentrations. Comparable results were obtained with Ksgn, even if no statistical significance was obtained on TEER with the lowest concentration of 0.1 µg/mL (Figure 2).

## 4. Conclusions

This study showed the effectiveness, at low concentrations, of Sangiovese *cv* pomace seeds extract-fortified kefir in a validated *in vitro* model of inflammation-impaired intestinal barrier. Nevertheless, to the best of our knowledge, this is the very first study on this new dairy formulation as well as one of first investigations on kefir activity on gut health. Thus, some limitations should be underlined and considered for future analysis and development. First, the different mechanistic role of antioxidant compounds of kefir and Sangiovese pomace in the final positive effect in the Caco-2 inflammatory model is worth to be better investigated. Moreover, the fascinating regulation of polyphenols metabolism by kefir fermentative process is another point to explore in a deeper way. Finally, this is a preliminary study which open the way for further studies to be conducted using more reliable *in vivo* models of intestinal inflammation.

## Figures and Tables

**Table 1:** Antioxidant activity of Sangiovese *cv* grape skins and seed extracts. TPE = total phenolic equivalent; TAC = total antioxidant capacity; GA = gallic acid; CT = catechin.

Sample	TPE (meq GA/g Extract)	TAC (meq CT/g Extract)	IC_50_ (mg ml^−1^)	
			DPPH Radical	ABTS Radical
**sgnbs**	0.549 ± 0.019	0.859 ± 0.018	0.160 ± 0.005	0.085 ± 0.001
**sgnss**	1.620 ± 0.054	0.520 ± 0.014	0.012 ± 0.001	0.004 ± 0.001

**Table 2:** ESI(+) mass spectrum of sgnss.

*m/z*	Formula [M + H]^+^	RDB	Error (ppm)	MS^2^	Tentative Identification
130.0863	C_6_H_12_NO_2_	1.5	0.1		Pipecolic acid
130.0498	C_5_H_8_N	2.5	−0.5		Oxoproline
189.0758	C_8_H_13_O_5_	2.5	0.1		
280.2636	C_18_H_34_NO	2.5	0.4		Too many possible compounds. Octadecadienamide or
284.2952	C_18_H_38_NO	0.5	1.5		Stearamide
302.3054	C_18_H_40_NO_2_	−0.5	0.1	MS^2^: 284.2948, 266.2819, 254.2834, 240.2679, 109.1007 C_8_H_13_^+^ 97.0993 C_7_H_13_^+^ 95.0841 C_7_H_11_^+^ 83.0859 C_6_H_11_^+^ 81.0689 C_6_H_9_^+^ 69.0683 C_5_H_9_^+^ 67.0538 C_5_H_7_^+^ 60.0435 C_2_H_6_ON^+^	Dihydrosphingosine or isomer
318.3006	C_18_H_40_NO_3_	−0.5	1.0		Phytosphingosine
331.2340	C_15_H_31_N_4_O_4_	2.5	0.1		Too many possible compounds
439.1387	C_24_H_23_O_8_	13.5	−0.1	MS^2^: 277.0891 (−162)	Flavonoid glycoside
463.1240	C_22_H_23_O_11_	11.5	1.1		Peonidin glucoside
493.1335	[C_23_H_25_O_12_]^+^	11.5	−1–1	MS^2^: 331, 315, 287,137	Malvidin-3-O-glucoside
639.1714	C_32_H_31_O_14_	17.5	0.9		Malvidin cumaroyl glucoside

RDB: rings and/or double bonds; MS^2^: tandem mass spectrometry.

**Table 3:** ESI(−) mass spectrum of sgnss.

*m/z*	Formula [M − H]^−^	RDB	Error (ppm)	MS^2^	Tentative Identification
133.0145	C_4_H_5_O_5_	2.5	1.9		Malic acid
149.0094	C_4_H_5_O_6_	2.5	1.6		Tartaric acid
163.0249	C_5_H_7_O_6_	2.5	0.5		2-Dehydro-D-xylonate
191.0198	C_6_H_7_O_7_	3.5	0.3		Citric acid
191.0561	C_7_H_11_O_6_	2.5	−0.2	MS^2^ 191: 173 (−18); 147 (−44 CO_2_); 136 (−58); 111 (−80); 85 (−106) MS^3^ 191->173: 155 (−18), 129 (−44), 111 (−62)	Quinic acid
193.0352	C_6_H_9_O_7_	2.5	−0.9		Glucuronic acid
193.0564	C_10_H_9_O_4_	6.5	0.1		Ferulic acid
205.0352	C_7_H_9_O_7_	3.5	−0.8		Acid methyl citric/Acid homocitric
207.0509	C_7_H_11_O_7_	2.5	−0.4		Methyl glucuronic acid
221.0666	C_8_H_13_O_7_	2.5	−0.3		Ethyl glucuronic acid
223.0460	C_7_H_11_O_8_	2.5	0.5	MS^2^ 223: 205 (−18); 191 (−32 CH_3_OH); 163 (−60); 131 (−92 (60 + 32)); 113 (131 − 18); 103 (131 − 28); 87 (−136: 131+44)) MS^3^: 223->131: 103 (−28); 87 (−44); 59(−(44 + 28)) MS^3^: 223->113: 85 (−28)	Hydroxymethyl monoglycosylpyranosonic acid
311.0622	C_10_H_15_O_11_	3.5	0.7		Glycosyl tartrate
359.0757	C_12_H_20_O_10_Cl	2.5	3.1		Anhydrodisaccharide

RDB: rings and/or double bonds; MS2: tandem mass spectrometry.

**Table 4:** Quantitative analysis of samples by ^1^H NMR. The SD is 3% of the measured amount.

Molecule	Kwht	Ksgn
**Amino Acids (mg L^−1^)**		
Valine	28.12	28.82
Isoleucine	17.84	20.59
Alanine	33.50	4.28
Tyrosine	9.78	9.24
Phenylalanine	22.80	40.20
**Carbohydrates (g L^−1^)**		
Galactose	4.22	10.72
Glucose	3.65	8.31
Lactose	0.0	2.26
**Organic Acids (mg L^−1^)**		
Lactic acid	1480	2610
2-hydroxy-isobutyrric acid	13.45	6.41
Acetic acid	7.21	10.09
Pyruvic acid	31.00	16.82
Succinic acid	21.72	2.83
Citric acid	278.96	1006
Fumaric acid	4.87	11.72
Formic acid	3.31	3.31

**Table 5:** Antioxidant activity of simple and fortified kefir. TPE = total phenolic equivalent; TAC = total antioxidant capacity; GA = gallic acid; CT = catechin.

Sample	TPE (meq GA/L)	TAC (meq CT/L)	IC_50_ (mg mL^−1^)
			DPPH Radical
**Kwht**	1.615 ± 0.005	0.558 ± 0.004	0.107 ± 0.001
**Ksgn**	1.975 ± 0.004	0.785 ± 0.003	0.090 ± 0.001
